# Comparison of Trust Assessment Scales Based on Item Response Theory

**DOI:** 10.3389/fpsyg.2020.00010

**Published:** 2020-01-23

**Authors:** Buyun Dai, Wenqing Zhang, Yang Wang, Xiaozhu Jian

**Affiliations:** ^1^School of Psychology, Jiangxi Normal University, Nanchang, China; ^2^School of Public Administration, Guangdong University of Finance, Guangzhou, China; ^3^School of Education, Jinggangshan University, Ji’an, China; ^4^Education Center for Mental Health, Jiangxi University of Applied Science, Nanchang, China

**Keywords:** interpersonal trust, college students, item response theory, scale evaluation, Chinese culture

## Abstract

Three widely used interpersonal trust measurement scales [Interpersonal Trust Scale (ITS), Philosophies of Human Nature Scale (RPHNS), Company Trust Scale (CTS)] have seldom been applied in non-Western contexts. Different social environments may lead to variation in the level or structure of trust. Therefore, it is necessary to compare the applicability of these scales to different levels of trust-related traits in Eastern cultures so that researchers can choose appropriate scales for relevant studies. This study attempted to conduct a comparative analysis of the ITS, RPHNS, and CTS. A sample of 725 Chinese college students was analyzed. Total score correlations and latent factor correlations estimated by confirmatory factor analysis (CFA) for a first-order three-factor model were assessed, and then the quality of the item parameters, test reliability and standard errors, and test information were assessed. The results are as follows: (1) the ITS and the RPHNS assessed almost the same trust traits; therefore, only the ITS and the RPHNS are compared in the next sections; (2) the original structure of only the RPHNS is verified; (3) some items on the ITS do not work well, while the RPHNS has higher overall test reliability; and (4) the average item information provided by the RPHNS is higher across all trait levels. In most cases, the RPHNS is the better choice in the Chinese cultural context.

## Introduction

Trust refers to a positive psychological expectation that an individual holds toward the behavior and purpose of someone he/she meets during his/her interactions with others or the social environment (Zhao et al., 2013). Research has proven that trust serves as a prerequisite for a sound relationship in social interactions (Righetti et al., 2011). In a cooperatively interactive group activity, trust is conducive to the consolidation of solidarity between group members and the enhancement of group performance (Stolle et al., 2008; Chen et al., 2010). In politics, trust is also one of the decisive factors that determines whether people support a public policy (Zhang et al., 2014). Economically, trust is helpful in simplifying transaction procedures and reducing transaction costs. However, trust may be the major cause of being tricked or duped (Shi et al., 2015). Studies have shown that there are relatively close relationships between interpersonal trust and personality, ego, depressive emotions, and Internet addiction among college students (Xin and Zhou, 2012; Xu T.J. et al., 2017).

In studies on interpersonal trust, widely used measuring tools include the Interpersonal Trust Scale (ITS) developed by Hochreich and Rotter (1970) based on social learning theory, the Philosophies of Human Nature Scale (RPHNS) revised by Wrightsman (1964), and the Company Trust Scale (CTS) created by Hunt et al. (1983). Although these three scales are widely used in Western countries, studies on their reliability and validity in a Chinese context are relatively few and insufficient (Jian, 2007). Additionally, changes in the social environment influence the level and structure of trust (Yang and Peng, 1999). Therefore, it is necessary to determine the factor structure and psychometric characteristics of these scales and to compare their applicability to different trust properties while accounting for the current period and the Chinese cultural context. This approach will allow researchers to choose appropriate scales and conduct related research in contemporary Chinese cultural contexts.

The aforementioned three scales were all created based on classical test theory (CTT). However, recent years have seen the rise of item response theory (IRT) and its technology. IRT has been used to evaluate the psychometric characteristics of three types of depression assessment scales (Adler et al., 2012; Umegaki and Todo, 2017). Compared with CTT, IRT has the following excellent properties. First, the category response threshold (parameter b) of the item and the trust property of the subject use the same metric system. Second, the results of different experiments with the same psychological traits can be compared (Luo, 2012). Third, through item parameter estimation, IRT can directly and accurately reflect the experimental characteristics of each item. Moreover, the application trend of the scale for different features can be demonstrated via a reliability curve and an average item information curve (Olino et al., 2013). Thus, the purpose of this study is to compare the psychometric characteristics of the aforementioned three scales via IRT technology and to make several suggestions for the application of the scales.

## Materials and Methods

### Participants

Students from four universities in Nanchang City completed the questionnaires. A total of 725 valid questionnaires were collected. The age of the subjects ranged from 17 to 23 years (*M* = 19.16, *SD* = 1.184). A total of 37.1% of the respondents were male, and 62.9% were female.

### Measures

The Chinese versions of the three trust assessment scales are applied in this study (revised edition; Wang et al., 1999).

The Chinese version of the ITS has 25 items with two dimensions: trust in relatives and friends and trust in people who have no direct relation. The aim is to measure subjects’ judgment of the reliability of others’ words and behavior. Scores are given on a five-point scale. There are 12 positive items and 13 negative items.

The Chinese version of the RPHNS has 20 items with two dimensions: trustworthiness and cynicism. Scores are given on a six-point scale ranging from −3 to 3. For the convenience of the IRT analysis, scores of one to six are given in this research. There are 10 positive items and 10 negative items.

The Chinese version of the CTS has 18 items with three dimensions: dependability, predictability, and reliability. The goal is to measure the degree to which intimates trust each other. Scores are given on a seven-point scale. There are 9 positive items and 9 negative items.

### Analysis

(1) Common method bias (CMB) test. Before analyzing the psychometric characteristics of the scales, Harman’s single-factor test was used to determine whether CMB existed (Zhou and Long, 2004).
(2) Analysis of trait congruency. To ensure that the same psychological properties were measured by all three scales, correlations among the total scores of the three scales were analyzed. On the basis of the three scales, a higher-order model included all dimensions in the three scales, treating a single scale as a second-order structure. In addition, confirmatory factor analysis (CFA) was employed to assess the latent correlation among all the potential factors to verify the congruency of the scales in terms of psychological properties (Umegaki and Todo, 2017).
(3) Construct validity analysis. These scales are widely used in the West and the East (Guinot et al., 2014; Jin et al., 2017). However, religious beliefs and social class problems affect these scales, meaning that they may not be adequately applicable in the Chinese cultural context. Moreover, few studies have explored the localized structure of these scales in China. Therefore, CFA was adopted to verify the original structures of the scales. If the original structure was not verified, exploratory factor analysis (EFA) was performed. Three fit indices, i.e., the comparative fit index (CFI), standardized root mean square residual (SRMR), and root mean square error of approximation (RSMEA), were employed for assessment purposes in both analytical methods.
(4) Analysis of item parameters and test information under the guidance of IRT. The common IRT multilevel score models are as follows: the generalized partial credit model (GPCM) (Muraki, 1992), the graded response model (GRM) (Samejima, 1969), and the generalized rating scale model (GRSM) (Masters, 1982). With the aim of selecting a model with a good fit to the test data, indices such as the Akaike information criterion (AIC), Bayesian information criterion (BIC), and −2 × Log-Lik were used to compare the fit precision of the scales under different IRT models. Once the model was determined, the psychometric characteristics of the scales were further investigated within the IRT framework. In addition, the scales’ item parameter quality, differential item functioning (DIF), reliability, deviation, average item information, and relative efficiency were analyzed, and all the scales were compared.

### Data Analysis: Software and Algorithm

SPSS 23.0 was used to execute the descriptive statistics analysis and reliability analysis. The maximum likelihood estimation was applied when conducting EFA and CFA in Mplus 7.4. The package mirt in R was employed to estimate the IRT parameters (the EM algorithm was used), and a model-fitting test was conducted. The package lordif in R was used in DIF analysis. Figures including the reliability curve and the standard error curve were created using the catR and plotrix packages in R.

## Results

### CMB Test

The CMB test result indicated that the characteristic roots of 18 factors exceeded 1, the first (largest) of which explained merely 10.76 percent of the total variance of the data, less than 40 percent of the critical value (Zhou and Long, 2004). Therefore, no CMB exists.

### Analysis of Trait Congruency

In this research, the Cronbach’s alpha coefficients of the ITS, RPHNS, and CTS were 0.70, 0.74, and 0.71, respectively, indicating acceptable reliability. The analysis of the correlation among the three scales revealed moderately positive correlations among the total scores of all the tests, with the following correlation results: 0.57 (ITS and RPHNS), 0.35 (ITS and CTS), and 0.39 (RPHNS and CTS), *P* < 0.01. A CFA model formulated to test the relativity among the three scales yielded the following fit indices: CFI = 0.66, SRMR = 0.08, and RMSEA = 0.04. For all three scales, the multidimensional structure led to a relatively low CFI value (Umegaki and Todo, 2017). The SRMR and RMSEA fit well with each other, indicating an effective model fit (Maydeu-Olivares et al., 2017). The correlations among the factors were 0.80 (ITS and RPHNS), 0.48 (ITS and CTS), 0.57 (RPHNS and CTS).

Based on the above results, the ITS and the RPHNS assessed almost the same trust traits, but the CTS may assessed different traits. To ensure the fairness and rigor of comparison, the next sections aim to determine the psychometric characteristics of only the ITS and the RPHNS and to compare their applicability to different trust properties.

A CFA model was established only for the ITS and the RPHNS, with the following results: CFI = 0.75, SRMR = 0.08, RSMEA = 0.06, and a factor correlation of 0.78.

### Construct Validity Analysis

Confirmatory factor analysis was used to confirm the original factor structures of the scales, and the results are shown in Table 1. According to the CFI, SRMR, and RMSEA fit indices, the original factor structure of the RPHNS fit well, but the original factor structure of the ITS was not verified. Even after the addition of a higher-order model, the results showed little change. As a result, it was essential to conduct new analyses of the structure and dimension of the ITS. The bi-factor model, oblique factor model and higher-order model were all considered, and the higher-order model was the final choice. The reasons for the selection of the higher-order model are as follows. First, from the perspective of model fit, although both the higher-order model and the bi-factor model fit well, because of the complexity of the latter model, it must estimate more parameters and is more difficult to converge (Xu S. et al., 2017). Second, the oblique factor model cannot analyze common effects. However, because a higher-order model is superior to a lower-order model, it separates common effects from unique effects and places more emphasis on the in-depth analysis of the factor structure (Gu and Wen, 2017). Finally, because comparison of the scales was the major purpose of this research, attention must be paid to the scales from an overall perspective.

**TABLE 1:** Scales’ fit indices for different structures.

	ITS			RPHNS		
Structure	CFI	SRMR	RMSEA	CFI	SRMR	RMSEA
Original structure	0.61	0.07	0.06	0.86	0.05	0.04
Higher-order model of the original structure	0.68	0.06	0.05	0.88	0.07	0.07
Re-exploration	0.91	0.04	0.03			
Higher-order verification	0.87	0.07	0.06			

The data were randomly divided into two equal parts, one of which was subjected to EFA and the other to CFA in the higher-order model. The fit indices of the structure of the scales and the factor loading of the higher-order model are shown in Table 1 and Supplementary Appendix 1, respectively. In Supplementary Appendix 1, most of the items have relatively high factor loadings, but there are some low values, as seen in the 18th, 23rd, and 24th items on the ITS, the 4th and 17th items on the RPHNS. Although the above items had rather low factor loadings, they were not deleted because revising the scales was not the purpose of this research.

Table 2 shows the comparison between the old and new structures in detail. (1) In contrast to its former two-dimensional structure, the ITS encompassed three dimensions. Based on the names of the previous dimensions and the factor loadings of the items, these three dimensions were named social phenomena, trust in others, and political trust. Table 2 indicates that political trust is the new ITS dimension. (2) The original dimensions of the RPHNS were verified.

**TABLE 2:** Comparison between the original and new dimensions of the ITS.

Original dimensions and items	New dimensions and items
Social phenomena	Social phenomena
1,2,3,4,7,8,11,12,13,14,15,18,24	1,2,4,5,7,10,11,15,17,19,24
Trust in others 5,6,9,10,16,17,19,20,21,22,23,25	Trust in others 6,8,9,14,16,18,20,22,23
	Political trust 3,12,13,21,25

### IRT Analysis

#### Fit Analysis of the IRT Model

A comparison of the fit precision of the two scales was performed within the frameworks of the GPCM, GRSM, and GRM. The indices compared were the AIC, BIC, and −2 × Log-Lik. As shown in Table 3, the GRM generally fit the two scales best and was therefore selected. The mathematical expression is as follows: $p_{i\,k}\,\left(\theta \right)=p_{i\,k}^{*}\,\left(\theta \right)-p_{i,k+1}^{*}\,\left(\theta \right)$, where “*i*” refers to the item number, and “*k*” refers to the scoring category. Furthermore, “$p_{i\,k}^{*}\,\left(\theta \right)=\frac{1}{1+e^{-1.7\cdot a_{i}\,\left(\theta -b_{i\,k}\right)}}$” refers to the probability of answering by a subject whose psychological properties level is “*θ*” and whose score is “*k*” or more on item “*i*” (Chen et al., 2006).

**TABLE 3:** Psychometric characteristics of the scales within the IRT framework.

	GPCM			GRSM			GRM		
	AIC	BIC	−2 × Log-Lik	AIC	BIC	−2 × Log-Lik	AIC	BIC	−2 × Log-Lik
ITS	48549.45	49133.80	48293.44	48744.22	48999.87	48632.22	48426.97	49011.32	48170.96
RPHNS	43857.42	44414.39	43613.42	43915.32	44217.57	43806.32	43636.28	44193.24	43392.28

#### Estimation and Analysis of the Item Parameters

The item parameters of the ITS, RPHNS were estimated and analyzed via the GRM model, detailed results of which are shown in Table 4. The underperforming items whose discrimination was less than 0.7 (Fliege et al., 2005) on these three scales were items 3, 8, 13, 17, 18, 21, 23, and 24 of the ITS and items 3, 8. Hence, with respect to item quality, the RPHNS was better than the other.

**TABLE 4:** Item parameters of the scales (under the GRM).

Item	a	b1	b2	b3	b4	b5
ITS-1	0.96	–2.08	0.69	1.96	3.63	
ITS-2	1.00	–2.15	0.08	1.58	3.66	
ITS-3	0.38	–9.64	–4.04	–1.03	2.49	
ITS-4	0.99	–3.28	–0.84	0.45	2.23	
ITS-5	0.83	–2.57	0.08	1.54	4.07	
ITS-6	0.84	–4.23	–1.87	–0.10	2.08	
ITS-7	0.72	–4.15	–1.83	–0.15	1.65	
ITS-8	0.59	–6.70	–2.03	0.18	3.37	
ITS-9	0.82	–1.92	0.85	2.54	5.03	
ITS-10	1.00	–2.29	0.03	1.45	3.93	
ITS-11	1.15	–2.05	–0.03	1.16	2.89	
ITS-12	1.48	–3.76	–1.67	–0.65	0.89	
ITS-13	0.68	–2.81	0.27	2.59	5.83	
ITS-14	1.14	–3.42	–1.03	0.56	2.72	
ITS-15	0.74	–4.64	–1.27	0.80	3.11	
ITS-16	0.83	–5.29	–1.89	0.50	3.60	
ITS-17	0.40	–7.64	–1.84	2.04	7.72	
ITS-18	0.43	–5.71	–0.43	3.00	8.84	
ITS-19	1.02	–3.30	–0.54	1.02	3.14	
ITS-20	0.87	–4.58	–1.66	0.43	3.80	
ITS-21	0.06	–25.88	6.15	27.34	67.55	
ITS-22	0.88	–4.26	–1.60	–0.01	2.22	
ITS-23	0.57	–5.12	–1.19	1.23	5.16	
ITS-24	0.29	–13.83	–4.30	3.64	13.13	
ITS-25	0.78	–4.90	–1.86	0.12	3.01	
RPHNS-1	1.12	–2.18	–0.49	0.81	1.55	2.92
RPHNS-2	1.12	–4.16	–2.03	–0.84	0.54	2.58
RPHNS-3	1.18	–3.66	–1.32	0.19	1.40	2.76
RPHNS-4	0.58	–4.99	–2.11	0.07	2.39	6.28
RPHNS-5	0.96	–3.37	–1.64	–0.19	1.38	3.41
RPHNS-6	1.17	–3.37	–1.63	–0.21	0.69	2.35
RPHNS-7	1.16	–3.59	–1.64	–0.40	0.96	3.07
RPHNS-8	1.26	–2.67	–0.94	0.23	1.16	2.53
RPHNS-9	1.35	–3.02	–1.77	–0.52	0.37	1.33
RPHNS-10	1.25	–3.74	–2.25	–1.06	–0.03	1.40
RPHNS-11	0.77	–3.30	–0.69	1.20	3.10	6.05
RPHNS-12	0.95	–4.27	–2.16	–0.66	0.92	3.05
RPHNS-13	1.49	–2.42	–1.07	–0.03	0.98	2.14
RPHNS-14	1.21	–3.17	–1.45	–0.15	1.06	2.73
RPHNS-15	1.28	–3.44	–1.81	–0.70	0.47	1.76
RPHNS-16	1.22	–2.79	–1.35	0.03	1.14	2.66
RPHNS-17	0.57	–7.44	–3.37	–0.90	1.82	5.95
RPHNS-18	1.75	–3.39	–1.81	–0.99	0.09	1.51
RPHNS-19	1.10	–3.60	–1.46	–0.07	1.07	2.44
RPHNS-20	1.31	–3.23	–1.75	–0.66	0.64	2.25

#### DIF Analysis

Region (urban, rural) and gender (male, female) were selected as grouping variables. The logistic regression (LR) method and McFadden’s pseudo R^2^ variation were used for DIF analysis. When R^2^ variation was greater than 0.02, DIF was indicated to exist in the item (Choi et al., 2010). The results showed that there was no DIF between the two scales.

#### Test Reliability Coefficient and Deviation Curve

**FIGURE 1:**
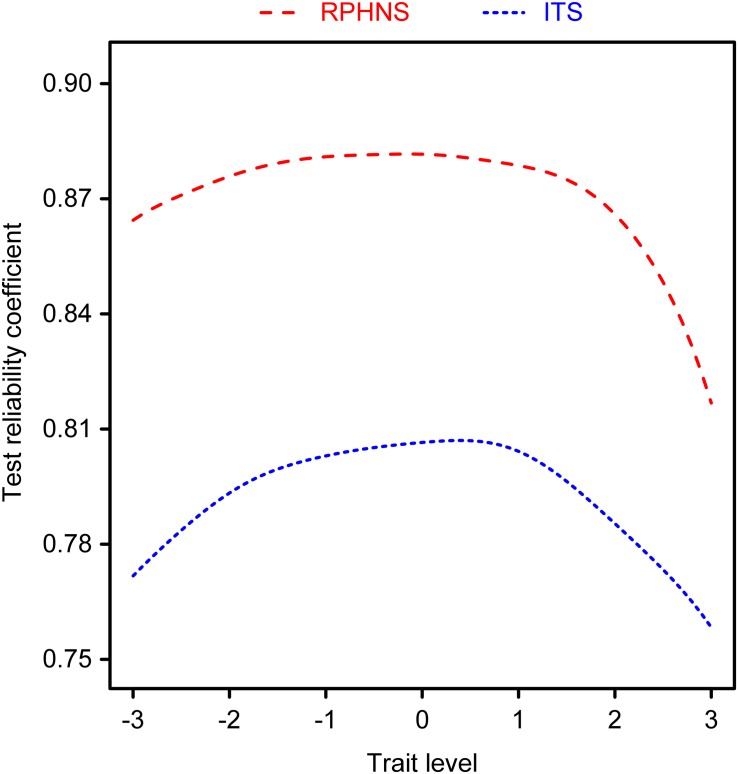
Reliability chart of scales by different trust properties.

**FIGURE 2:**
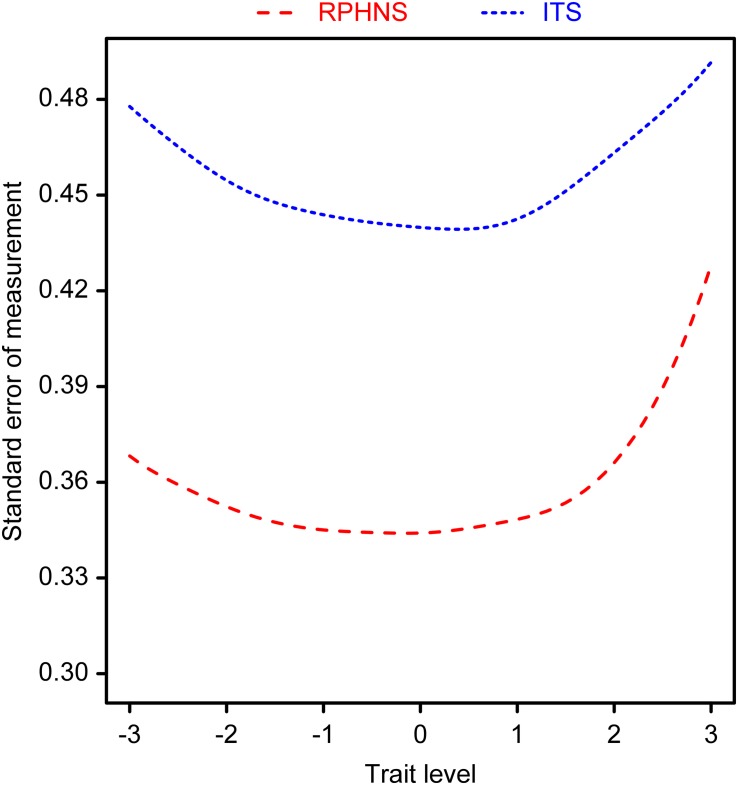
Deviation chart of scales by different trust properties.

One of the advantages of IRT is that it can offer every subject a corresponding test reliability and deviation. The formula for calculating the reliability coefficient is $r_{x\,x}\,\left(\theta \right)=1-\frac{1}{I\,\left(\theta \right)}$, and the formula for deviation is $S\,E\,\left(\theta \right)=\frac{1}{\sqrt{I\,\left(\theta \right)}}$. “*I*(θ)” refers to the amount of information that a subject whose psychological properties level is *θ* contributes to the test (Luo et al., 2009). The reliability and deviation of the two scales were calculated under the GRM model, and the results are shown in Figures 1, 2, respectively. The ITS test reliability coefficients of subjects with different trust properties ranged from 0.76 to 0.80. Subjects whose trust properties ranged from −1 to 1 yielded test reliability values of approximately 0.8. However, the reliability values of subjects whose trust properties were at the two extremes were relatively low. The test reliability coefficients of the RPHNS ranged from 0.82 to 0.88. Subjects whose trust properties ranged from −2 to 2 yielded test reliability values of approximately 0.87. Likewise, the test reliability coefficients of subjects whose trust properties were lower than two standard deviations of the average value were approximately 0.86. However, the test reliability coefficients of subjects whose trust properties were higher than 2 standard deviations of the average value decreased to approximately 0.84. Generally, the RPHNS exhibited comparably high test reliability and guaranteed the test reliability of subjects with relatively low trust property levels.

#### Average Item Information Curve

**FIGURE 3:**
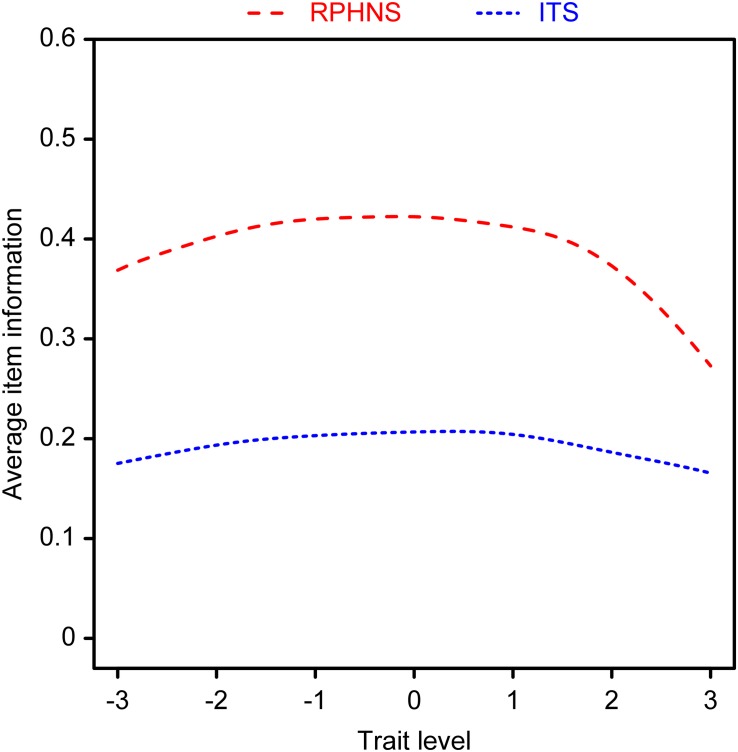
Average item information curve.

The average item information curve is the amount of information that each item offers. As shown in Figure 3, the curves for each scale were in distinct locations. At different trait levels, the curve of the RPHNS was always above the curve of the ITS, which indicated that the RPHNS had higher item quality.

## Discussion

As mentioned above, although the CTS was significantly positively correlated with the other two scales in terms of total scores, the correlation coefficient was somewhat low. The total scores correlation coefficient between the RPHNS and ITS was 0.57. The CFA results show that the factors were highly correlated with one another, with a correlation coefficient of 0.80 (After removing the CTS, the factor correlation between them was 0.78). By comparison, the correlation coefficients of the CTS with respect to both scores and factors ranged from 0.48 to 0.57. Both the ITS and RPHNS involve commitment to moral standards and beliefs and have similar item content. For the trusted subject reflected in the scale, the CTS focuses on familiar subjects, such as trust in companions’ behavior and relationship. The RPHNS and ITS measure trust in general subjects in society or in the social environment. For example, the ITS addresses trust in the courts, officers, sales promoters, and experts, and the ITS and RPHNS evaluate most people’s trust in attitudes toward social life. Therefore, such differences in focus may lead to low correlation coefficients.

According to the construct validity analysis, the original dimensions of only the RPHNS were verified. A three-dimensional construct was verified after re-exploring the ITS. The factor of political trust was added to the original dimensions. The scores for the ITS suggest disparities in terms of religious belief, family background, and social class (Xu, 2010). With regard to the property of trust, the related research in the West places more emphasis on personal factors, including responsibility and ability (such as trust in general subjects in society in the ITS), while Chinese studies pay more attention to interpersonal relations, including personal relations in the process of socialization (Chang and Holt, 1991). Differences also exist between China and Western countries in the relationship between personal factors and interpersonal relations. In the West, personal factors are independent of human relations and sometimes are prior to the latter (Wang, 2008). However, in Chinese culture, when Chinese people communicate with others, they have different modes of trust because of their different social identities, statuses and relationships (Wang et al., 2016). Hence, deviation is unavoidable if the Western scales are directly applied to Chinese subjects. Furthermore, trust, interpersonal relations, and social intercourse are often intertwined. Changes in the social environment have an uncertain impact on trust. More specifically, the level or the structure of trust may be influenced (Yang and Peng, 1999).

The analytical results of IRT showed that although the same psychological properties were tested by the ITS and the RPHNS, different psychometric characteristics appeared during the application of the scales. In terms of item parameters, some items of the ITS did not work well due to their low discrimination. This finding is in accordance with previous research results: scores on items that convey negative meanings are poorly correlated with the total scores for the ITS (Jian, 2007). In addition, some items concerning politics and beliefs also showed low discrimination, including item 3 (national prospects), item 13 (international affairs), and item 18 (firm belief) of the ITS and item 4 (principles of treating others in the Bible) and item 17 (adherence to ideas) of the RPHNS. Regarding test reliability, the RPHNS showed high test reliability coefficients. Even the subjects whose trust properties were low had high test reliability.

## Conclusion and Suggestions

In this study, the psychometric characteristics of two common trust assessment scales were analyzed and compared using college students as the research sample. The following results were found: the ITS and the RPHNS assessed almost the same trust traits, while the CTS did not; both the ITS and the RPHNS had high reliability, with higher reliability exhibited by the RPHNS; and the original dimensions of only the RPHNS were verified, while a new dimension was added (i.e., political trust) after the re-exploration and verification of the ITS.

Based on the conclusions above, some suggestions are provided for selecting the best trust assessment scale. First, taking the properties of subjects into consideration, trust can be divided into general trust and special trust. The former refers to trust in all those who share the same beliefs, while the latter refers to trust in intimate people only, such as relatives and friends, in the process of socialization (Wessen et al., 1951). Regarding the purpose of the scale creators and the item content of the scales, the RPHNS can be used to test general trust (Jian and Tang, 2006); the ITS is the better choice to test both general and special trust. Second, the RPHNS should be used when a rigorous scale structure is needed because its structure is stable even if uncertain changes in the social environment influence the level and structure of trust. Third, the RPHNS is adequate when there is high demand for test reliability and item quality.

### Limitations and Outlook

As a psychological property, trust differs by gender. Females display a higher level of trust than males (Li et al., 2007). Candidly, gender balance was not sufficiently achieved in this study. This limitation may have caused an imbalance in the subjects’ trust properties in this research. In terms of sample representativeness and result generalizability, this study has certain deficiencies. However, although the study was conducted in only one area, the college students who were tested came from all parts of China. Moreover, the sampled students covered the junior college and undergraduate education levels, taking urban and rural areas and grade level into account. To a certain extent, therefore, this sample is representative of Chinese college students overall.

This study repeated the analysis of the three scales (one of which was abandoned during the process of data analysis). From the perspective of cross-cultural comparison and research rigor, there are several reasons for these shortcomings. (1) The multidimensionality of the trust scales increases the workload of data analysis and increases the difficulty of construct verification as well as comparative analysis, which is embodied in the analysis of trait congruency. In addition, the multidimensional construct is more complex in the model, and more parameters must therefore be used. To ensure the accuracy of the results, we have used a variety of analytical methods to improve the quality of this research. (2) The cross-cultural context makes construct verification more complex. This difficulty also shows that cross-cultural research is necessary and that it requires more rigorous, high-quality research. (3) To sum up, to achieve balance between the research purpose and the accuracy of the results, this study focuses more on the analysis of the scales.

Three popular foreign scales were selected because they are widely used. However, according to previous research, cultural disparity exists between China and Western countries with respect to trust properties. Thus, deviation is unavoidable when Western scales are applied to Chinese subjects. Future researchers should create a new trust assessment scale that reflects the Chinese cultural context.

## Data Availability Statement

All datasets generated for this study are included in the article/Supplementary Material.

## Ethics Statement

The studies involving human participants were reviewed and approved by the Center of Mental Health Education and Research, School of Psychology, Jiangxi Normal University. The patients/participants provided their informed consent to participate in this study.

## Author Contributions

BD and WZ collaborated on the design of the study and were responsible for the statistical analyses and the writing of the manuscript. YW provided ideas for data analysis. XJ provided ideas for manuscript writing. All authors drafted the manuscript and approved it for publication.

## Conflict of Interest

The authors declare that the research was conducted in the absence of any commercial or financial relationships that could be construed as a potential conflict of interest.

## References

[B1] Adler M. Hetta J. Isacsson G. Brodin U. (2012). An item response theory evaluation of three depression assessment instruments in a clinical sample. *BMC Med. Res. Methodol.* 12:84. 10.1186/1471-2288-12-84 22721257 PMC3599629

[B2] Chang H. C. Holt G. R. (1991). More than relationship: Chinese interaction and the principle of Kuan-Hsi. *Commun. Q.* 39 251–271. 10.1080/01463379109369802

[B3] Chen P. Ding S. L. Lin H. J. Zhou J. (2006). Item selection strategies of computerized adaptive testing based on graded response model. *Acta Psychol. Sin.* 38 461–467.

[B4] Chen Y. Shi K. Luo D.-X. (2010). Trust in organizations: maintaining and repair. *Adv. Psychol. Sci.* 18 664–670.

[B5] Choi S. W. Grady M. W. Dodd B. G. (2010). A new stopping rule for computerized adaptive testing. *Educ. Psychol. Meas.* 70 37–53. 10.1177/0013164410387338 21278821 PMC3028267

[B6] Fliege H. Becker J. Walter O. B. Bjorner J. B. Klapp B. F. Rose M. (2005). Development of a computer-adaptive test for depression (D-CAT). *Qual. Life Res.* 14 2277–2291. 10.1007/s11136-005-6651-9 16328907

[B7] Gu H. Wen Z. (2017). Reporting and interpreting multidimensional test scores: a bi-factor perspective. *Psychol. Dev. Educ.* 33 504–512. 10.16187/j.cnki.issn1001-4918.2017.04.15

[B8] Guinot J. Chiva R. Roca-Puig V. (2014). Interpersonal trust, stress and satisfaction at work: an empirical study. *Pers. Rev.* 43 96–115. 10.1108/pr-02-2012-0043

[B9] Hochreich D. J. Rotter J. B. (1970). Have college students become less trusting? *J. Personal. Soc. Psychol.* 15 211–214. 10.1037/h0029433 23504267

[B10] Hunt R. W. Kohn P. M. Mallozzi C. B. (1983). Factor analysis of the interpersonal trust scale with a noncollege population. *J. Personal. Assess.* 47 507–508. 10.1207/s15327752jpa4705_10 16367567

[B11] Jian J. (2007). *The Reliability and Validity of Related Scales of Interpersonal Trust and the Relations with Mental Health in Collegian.* master thesis, Shandong University, Jinan.

[B12] Jian J. Tang M. Q. (2006). The reliability and validity of revised philosophies of human nature scale in Chinese college students. *Chin. J. Clin. Psychol.* 14 347–348.

[B13] Jin X. Li Y. M. Li X. S. Yang L. Q. Lao Y. C. (2017). Relationship among online social attitude, online trust, interpersonal trust, social anxiety and loneliness. *Chin. J. Clin. Psychol.* 25 185–187. 10.16128/j.cnki.1005-3611.2017.01.042

[B14] Li H. J. Xu Y. Guo Y. Y. (2007). Interpersonal trust-distrust: is it unidimensional or bidimensional? *Psychol. Dev. Educ.* 23 112–116.

[B15] Luo Z. S. (2012). *Item Response Theory.* Beijing: Beijing Normal University Press.

[B16] Luo Z.-S. Ouyang X.-L. Qi S.-Q. Dai H.-Q. Ding S.-L. (2009). IRT information function of polytomously scored items under the graded response model. *Acta Psychol. Sin.* 40 1212–1220. 10.3724/sp.j.1041.2008.01212

[B17] Masters G. N. (1982). A rasch model for partial credit scoring. *Psychometrika* 47 149–174. 10.1007/bf02296272

[B18] Maydeu-Olivares A. Shi D. Rosseel Y. (2017). Assessing fit in structural equation models: a monte-carlo evaluation of rmsea versus srmr confidence intervals and tests of close fit. *Struct. Equat. Modeling Multidiscip. J.* 25 389–402. 10.1080/10705511.2017.1389611

[B19] Muraki E. (1992). A generalized partial credit model: application of an EM algorithm. *Appl. Psychol. Meas.* 16 159–176. 10.1177/014662169201600206

[B20] Olino T. M. Yu L. McMakin D. L. Forbes E. E. Seeley J. R. Lewinsohn P. M. (2013). Comparisons across depression assessment instruments in adolescence and young adulthood: an item response theory study using two linking methods. *J. Abnorm. Child Psychol.* 41 1267–1277. 10.1007/s10802-013-9756-6 23686132 PMC3795839

[B21] Righetti F. Finkenauer C. Rusbult C. (2011). The benefits of interpersonal regulatory fit for individual goal pursuit. *J. Pers. Soc. Psychol.* 101 720–736. 10.1037/a0023592 21534700

[B22] Samejima F. (1969). Estimation of latent ability using a response pattern of graded scores. *Psychometrika* 34 1–97. 10.1007/BF03372160 27519776

[B23] Shi Y. Xu F. Luo J. Li Y. Liu C. (2015). Trust in behavioral economics: formation mechanisms and influential factors. *Adv. Psychol. Sci.* 23 1236–1244. 10.3724/sp.j.1042.2015.01236

[B24] Stolle D. Soroka S. Johnston R. (2008). When does diversity erode trust? Neighborhood diversity, interpersonal trust and the mediating effect of social interactions. *Polit. Stud.* 56 57–75. 10.1111/j.1467-9248.2007.00717.x 15748676

[B25] Umegaki Y. Todo N. (2017). Psychometric properties of the Japanese CES–D, SDS, and PHQ–9 depression scales in university students. *Psychol. Assess.* 29 354–359. 10.1037/pas0000351 27322202

[B26] Wang D. D. (2008). *Research on Network Interpersonal Trust of College Students.* Doctoral Dissertation, East China Normal University, Shanghai.

[B27] Wang P. Liang Y. Li Y. Liu Y. (2016). The effects of characteristic perception and relationship perception on interpersonal trust. *Adv. Psychol. Sci.* 24 815–823. 10.3724/sp.j.1042.2016.00815 17100505

[B28] Wang X. D. Wang X. L. Ma H. (1999). *Mental Health Rating Scale Manual.* Beijing: China Journal of Mental Health Press.

[B29] Wessen A. F. Weber M. Gerth H. H. (1951). The religion of China: confucianism and taoism. *Am. Sociol. Rev.* 16 890–891. 10.2307/2087534

[B30] Wrightsman L. S. (1964). Measurement of philosophies of human nature. *Psychol. Rep.* 14 743–751. 10.2466/pr0.1964.14.3.743

[B31] Xin Z.-Q. Zhou Z. (2012). A cross-temporal meta-analysis of changes in Chinese college students’ interpersonal trust. *Adv. Psychol. Sci.* 20 344–353. 10.3724/sp.j.1042.2012.00344

[B32] Xu H. Y. (2010). Revision of interpersonal trust scale and its application in college students. *Data Cult. Educ.* 32 222–223.

[B33] Xu S. Yu Z. Li Y. (2017). Simulated data comparison of the predictive validity between bi-factor and high-order models. *Acta Psychol. Sin.* 49 1125–1136. 10.3724/sp.j.1041.2017.01125

[B34] Xu T. J. Hu P. Guo X. M. (2017). The Effect of social exclusion on interpersonal trust: the role of emotional cues. *Chin. J. Clin. Psychol.* 25 1064–1068.

[B35] Yang Z. Peng S. (1999). Conceptualization of interpersonal trust in China: a perspective of interpersonal relationship. *Sociol. Study* 13 3–23.

[B36] Zhang S. Xu Z. Xu Y. (2014). Social justice and political trust: the mechanism of cooperation with government. *Adv. Psychol. Sci.* 22 588–595. 10.3724/sp.j.1042.2014.00588

[B37] Zhao J. Sun X. Zhou Z. Wei H. Niu G. (2013). Interpersonal trust in online communication. *Adv. Psychol. Sci.* 21 1493–1501. 10.3724/sp.j.1042.2013.01493

[B38] Zhou H. Long L. (2004). Statistical remedies for common method biases. *Adv. Psychol. Sci.* 12 942–950. 10.3969/j.issn.1671-3710.2004.06.018

