# Supplementary material for: Comparison of Trust Assessment Scales Based on Item Response Theory
Source: Front Psychol. 2020 Jan 23;11:10. doi: 10.3389/fpsyg.2020.00010 (PMC6989430; doi:10.3389/fpsyg.2020.00010)
Supplement: Supplementary file 1 [file Data_Sheet_1.docx]

**Appendix 1.** Scales’ Factor Loadings in the Higher-Order Analysis

| Item | F1 | F2 | F3 | H |
| --- | --- | --- | --- | --- |
| RPHNS-1 | 0.58 |  |  |  |
| RPHNS-3 | 0.55 |  |  |  |
| RPHNS-6 | 0.55 |  |  |  |
| RPHNS-8 | 0.61 |  |  |  |
| RPHNS-9 | 0.58 |  |  | 0.39 |
| RPHNS-11 | 0.32 |  |  |  |
| RPHNS-13 | 0.51 |  |  |  |
| RPHNS-15 | 0.55 |  |  |  |
| RPHNS-16 | 0.54 |  |  |  |
| RPHNS-19 | 0.43 |  |  |  |
| RPHNS-2 |  | 0.40 |  |  |
| RPHNS-4 |  | 0.21 |  |  |
| RPHNS-5 |  | 0.42 |  |  |
| RPHNS-7 |  | 0.51 |  |  |
| RPHNS-10 |  | 0.53 |  |  |
| RPHNS-12 |  | 0.40 |  | 0.83 |
| RPHNS-14 |  | 0.52 |  |  |
| RPHNS-17 |  | 0.26 |  |  |
| RPHNS-18 |  | 0.61 |  |  |
| RPHNS-20 |  | 0.61 |  |  |
| ITS-1 | 0.48 |  |  |  |
| ITS-2 | 0.52 |  |  |  |
| ITS-4 | 0.40 |  |  |  |
| ITS-5 | 0.46 |  |  |  |
| ITS-7 | 0.35 |  |  |  |
| ITS-10 | 0.43 |  |  | 0.38 |
| ITS-11 | 0.49 |  |  |  |
| ITS-15 | 0.32 |  |  |  |
| ITS-17 | 0.37 |  |  |  |
| ITS-19 | 0.47 |  |  |  |
| ITS-24 | 0.10 |  |  |  |
| ITS-6 |  | 0.47 |  |  |
| ITS-8 |  | 0.35 |  |  |
| ITS-9 |  | 0.39 |  |  |
| ITS-14 |  | 0.32 |  |  |
| ITS-16 |  | 0.38 |  | 0.59 |
| ITS-18 |  | 0.13 |  |  |
| ITS-20 |  | 0.36 |  |  |
| ITS-22 |  | 0.35 |  |  |
| ITS-23 |  | 0.18 |  |  |
| ITS-3 |  |  | 0.35 |  |
| ITS-12 |  |  | 0.55 |  |
| ITS-13 |  |  | 0.38 | 0.62 |
| ITS-21 |  |  | 0.39 |  |
| ITS-25 |  |  | 0.37 |  |
